# Supplementary material for: Comparative Plastomes of Curcuma alismatifolia (Zingiberaceae) Reveal Diversified Patterns among 56 Different Cut-Flower Cultivars
Source: Genes (Basel). 2023 Aug 31;14(9):1743. doi: 10.3390/genes14091743 (PMC10531169; doi:10.3390/genes14091743)
Supplement: Supplementary file 1 [file genes-14-01743-s001.zip › Supplemental Figure S1.pdf]

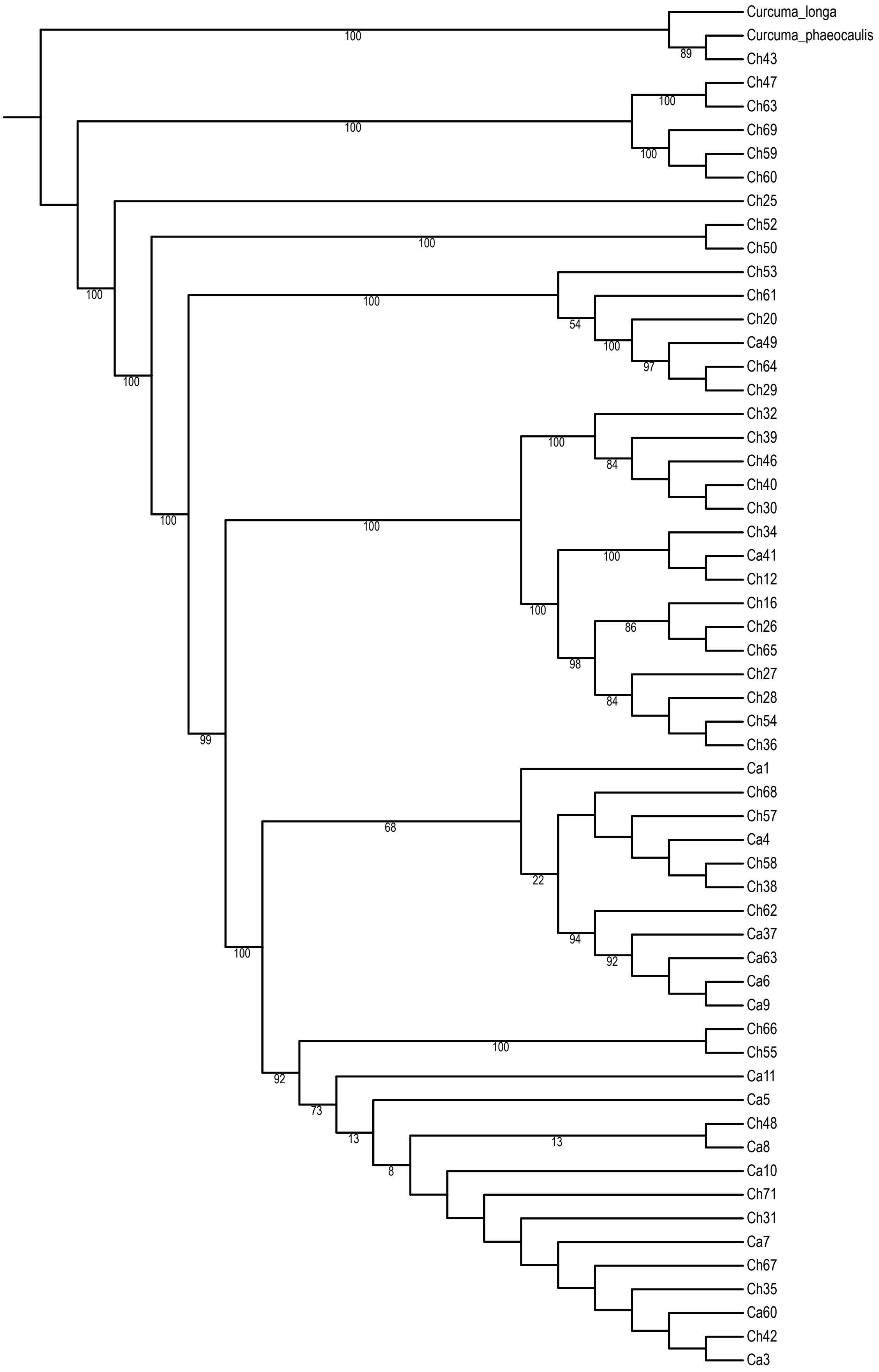


Figure S1. Phylogenetic tree of *C. alismatifolia* constructed on the CDS of 79 genes that code for pro teins. Numbers at nodes indicate the ultrafast bootstrap values generated by IQ-TREE.
